# Real-Time PCR Improves *Helicobacter pylori* Detection in Patients with Peptic Ulcer Bleeding

**DOI:** 10.1371/journal.pone.0020009

**Published:** 2011-05-20

**Authors:** María José Ramírez-Lázaro, Sergio Lario, Alex Casalots, Esther Sanfeliu, Loreto Boix, Pilar García-Iglesias, Jordi Sánchez-Delgado, Antònia Montserrat, Maria Rosa Bella-Cueto, Marta Gallach, Isabel Sanfeliu, Ferran Segura, Xavier Calvet

**Affiliations:** 1 Digestive Diseases Department, Hospital de Sabadell, Institut Universitari Parc Taulí, Departament de Medicina, Universitat Autònoma de Barcelona, Sabadell, Spain; 2 Centro de Investigación Biomédica en Red de Enfermedades Hepáticas y Digestivas (CIBERehd), Instituto de Salud Carlos III, Barcelona, Spain; 3 Pathology Department, Hospital de Sabadell, Sabadell, Spain; 4 BCLC Group, Liver Unit, Hospital Clínic de Barcelona, Barcelona, Spain; 5 Infectious Disease Department, Hospital de Sabadell, Institut Universitari Parc Taulí, Departament de Medicina, Universitat Autònoma de Barcelona, Sabadell, Spain; 6 Spanish Network for the Research in Infectious Diseases (REIPI RD06/0018), Sevilla, Spain; Fundació Institut Germans Trias i Pujol; Universitat Autònoma de Barcelona CibeRES, Spain

## Abstract

**Background and Aims:**

Histological and rapid urease tests to detect *H. pylori* in biopsy specimens obtained during peptic ulcer bleeding episodes (PUB) often produce false-negative results. We aimed to examine whether immunohistochemistry and real-time PCR can improve the sensitivity of these biopsies.

**Patients and Methods:**

We selected 52 histology-negative formalin-fixed paraffin-embedded biopsy specimens obtained during PUB episodes. Additional tests showed 10 were true negatives and 42 were false negatives. We also selected 17 histology-positive biopsy specimens obtained during PUB to use as controls. We performed immunohistochemistry staining and real-time PCR for 16S rRNA, ureA, and 23S rRNA for *H. pylori* genes on all specimens.

**Results:**

All controls were positive for *H. pylori* on all PCR assays and immunohistochemical staining. Regarding the 52 initially negative biopsies, all PCR tests were significantly more sensitive than immunohistochemical staining (p<0.01). Sensitivity and specificity were 55% and 80% for 16S rRNA PCR, 43% and 90% for ureA PCR, 41% and 80% for 23S rRNA PCR, and 7% and 100% for immunohistochemical staining, respectively. Combined analysis of PCR assays for two genes were significantly more sensitive than ureA or 23S rRNA PCR tests alone (p<0.05) and marginally better than 16S rRNA PCR alone. The best combination was 16S rRNA+ureA, with a sensitivity of 64% and a specificity of 80%.

**Conclusions:**

Real-time PCR improves the detection of *H. p*yl*ori* infection in histology-negative formalin-fixed paraffin-embedded biopsy samples obtained during PUB episodes. The low reported prevalence of *H. pylori* in PUB may be due to the failure of conventional tests to detect infection.

## Introduction

Peptic ulcer bleeding (PUB) is one of the severest complications of *Helicobacter pylori* infection [1]. Eradication therapy effectively prevents recurrent PUB [2]. However, the reported prevalence of *H. pylori* infection in bleeding ulcers is lower than in nonbleeding ulcers [3]. As tests for *H. pylori* are less reliable in the setting of PUB [4], whether this lower prevalence is genuine or merely reflects the lack of sensitivity of the diagnostic tests is unknown.

Moreover, indirect data suggest that conventional tests may also fail to detect *H. pylori* in other settings; for example, *H. pylori* “negative” MALT lymphomas have been reported to heal after eradication treatment [5]. In one study of the bacterial microbiota in the human stomach, polymerase chain reaction (PCR) detected *H. pylori* infection in gastric samples from 19 of 23 subjects; however, only 12 of these were positive on conventional tests [6]. Thus, highly sensitive molecular approaches might help detect *H. pylori* in settings such as PUB, gastric cancer, or MALT lymphoma in which *H. pylori* diagnosis is important but elusive.

Real-time PCR is a sensitive, accurate method of diagnosing *H. pylori* infection. Compared to other available methods for diagnosing *H. pylori* for clinical and research purposes, PCR yields high sensitivity and specificity for *H. pylori* in frozen samples from patients with nonbleeding peptic ulcer [7], [8]. PCR is also far better than histology at detecting *H. pylori* in fresh frozen gastric biopsies from PUB patients [9].

Although fresh or frozen biopsy specimens are the samples of choice for nucleic acid extraction, they require specialized storage and are rarely obtained in clinical practice. Formalin-fixed paraffin-embedded (FFPE) samples are the most widely available material for retrospective clinical studies. Given their potential for genomic analysis, these tissues represent an invaluable resource for research. DNA extracted from FFPE samples can also be used as a target for PCR: several experiments have confirmed that amplifying small PCR products is highly efficient and specific for detecting *H. pylori* [10], [11]. In this context, PCR for *H. pylori* in FFPE biopsies has proven reasonably accurate [11], [12]. Nonetheless, the best genes to detect *H. pylori* infection in FFPE biopsies have not been established. Potential candidates are 16S ribosomal RNA (16S rRNA) [13], ureaseA (ureA) [14], and 23S ribosomal RNA (23S rRNA) genes [15].

In a previous study, we found that biopsies obtained during the initial endoscopy were negative for *H. pylori* at histology in 25% of patients (58 of 232) with PUB. However, 82% of the 52 patients who underwent a delayed [^13^C]-urea breath test (UBT) four to eight weeks after bleeding had evidence of infection, so the final prevalence of *H. pylori* infection approached 100% [16]. Other authors have reported similar findings on delayed tests [17]–[19], confirming the low sensitivity and negative predictive values of conventional *H. pylori* tests during acute upper gastrointestinal bleeding. Overall, these studies suggest that “idiopathic” bleeding ulcers may be less common than previously reported [3].

The aim of the present study was to evaluate the potential usefulness of real-time PCR for improving detection of *H. pylori* infection in false-negative FFPE samples from patients with PUB. A secondary objective was to compare the sensitivity of different PCR targets and immunohistochemical staining for the detection of *H. pylori*.

## Methods

### Ethics Statement

The Ethics Committee of the Hospital de Sabadell approved the research protocol. Samples of control patients were prospectively obtained and both positive and negative control patients gave written informed consent to participate in the study. Regarding PUB cases, the patients were identified from a retrospective study and the samples obtained from the paraffin-embedded biopsy samples stored in the Pathology Department of the Hospital. In these patients, informed consent was not obtained in accordance with the Spanish Law 14/2007 of Biomedical Research. According to the law, archive samples may be used for biomedical research without informed consent when obtaining informed consent is not possible or extremely difficult, provided that a) the investigation performed is of general interest, b) there is no previous declaration of the patient against the use of the samples for research, c) data confidentiality is granted and d) the Ethics Committee of the Hospital evaluates and approves the study protocol.

### Patients

We initially used histology to select *H. pylori*-negative antral biopsy specimens obtained during acute PUB during emergency endoscopy in 58 patients; six patients did not complete follow-up and their samples were excluded from the analysis. Of the remaining 52 patients, 42 were positive and 10 were negative for *H. pylori* on a [^13^C]-UBT performed four to eight weeks after PUB. Antibiotics were stopped at least four weeks and proton pump inhibitors (PPI) at least two weeks before the [^13^C]-UBT. We included 17 histologically positive samples from patients with PUB as positive controls. This 69 patients (52 men and 17 women; mean age, 61.5±15.8 years; range, 22–90 years) were included between January 1995 and September 2000. They were selected from a large retrospective series of PUB patients followed up at the Digestive Diseases Department of the Hospital de Sabadell [16]. All patients were administered intravenous omeprazole after admission and underwent urgent upper gastrointestinal endoscopy in the first 24 hours to diagnose and treat the bleeding. All the biopsy samples analyzed in this study were obtained in this initial procedure. Table 1 shows the characteristics of the patients included in the study.

**Table 1:** Characteristics of PUB patients.

	*Hp* (+) patients (n = 17)	*Hp* (−) patients (n = 52)	*Hp* (−) controls (n = 32)
**Gender (Male/Female) (n)**	16/1	36/16	13/19
**Age (Years)**	53.4±14	64.2±15	45.8±16
**Endoscopic diagnosis (n)**			
Duodenal ulcer	14	26	-
Gastric ulcer	2	24	-
Erosive gastritis/duodenitis	1	2	-
**Blood in stomach (yes/no/not specified)**	5/12/0	6/43/3	-

We also selected 32 negative controls from a larger series of patients with dyspeptic symptoms who underwent endoscopy between 2006 and 2009 [20], [21]. In all patients, PPIs were avoided for at least two weeks and antibiotics stopped at least four weeks before endoscopy. To ensure that controls were truly uninfected, we included only those with strictly normal histological findings, including the absence of indirect histological signs of *H. pylori* infection such as lymphoid follicles or acute or chronic gastritis; moreover, all controls had no evidence of *H. pylori* on the rapid urease, UBT, and fecal tests.

### DNA Extraction

We retrieved FFPE gastric biopsy specimens collected at initial endoscopy from the Pathology Department's archives. Three 10 µm-thick sections were cut from each specimen and placed in a 1.5 ml polypropylene tube (Eppendorf, Hamburg, Germany). Every paraffin block contained from one to five antral biopsies. DNA was extracted as described by Chomczynski and Sacchi [22]. Briefly, paraffin was removed by washing three times with 1 ml of xylene for 5 minutes, followed by rehydration through three washes with 100% ethanol and two washes with 70% ethanol. After each step, the tissue was collected by centrifugation at 16000× g for 3 minutes. After the final 70% ethanol wash, the pellet was resuspended in 300 µl of lysis buffer containing 100 mmol/L sodium chloride, 10 mmol/L Tris/HCl (pH 8.0), 25 mmol/L ethylenediaminetetraacetic acid (EDTA), 0.5% sodium dodecyl sulfate (Applichem, Darmstadt, Germany), and 100 µg/ml proteinase K (Roche Applied Science, Mannheim, Germany) and incubated at 50°C overnight until tissue was completely solubilized. DNA was purified by phenol/chloroform/isoamyl alcohol (25∶24∶1, vol/vol/vol) followed by precipitation with 2.5 volumes of isopropanol in the presence of 0.1 volumes of 3 mol/L sodium acetate and 5 µg of linear acrylamide (Ambion, Austin, USA). The DNA pellet was washed once in 70% ethanol, dried, and resuspended in 50 µl of 10 mmol/L Tris/HCl (pH 8), 0.1 mmol/L EDTA.

### Bacterial strains

To evaluate the specificity of the PCR assays for 16S rRNA, ureA, and 23S rRNA, 11 bacterial species were grown on Columbia blood agar supplemented with 5% sheep blood (Biomerieux, Spain) at 37°C under appropriate atmospheric conditions. Bacterial species were obtained from clinical isolates (*Staphylococcus epidermidis*, *Corynebacterium sp.*, *Proteus sp.*, *Candida albicans*, and *Neisseria meningitidis)* or from the American Type Culture Collection (ATCC) (Rockville, MD, USA): *Pseudomonas aeruginosa*, *Enterococcus faecalis*, *Escherichia coli*, *Staphylococcus aureus*, *Streptococcus pneumoniae* and *Haemophilus influenzae*), and *Helicobacter pylori* strains J99 and 26695 (Table 2).

**Table 2:** Species specificity of PCR for *H. pylori*.

Bacterial species	Presence (+) or absence (−) of the following PCR product		
	16S rRNA	ureA	23S rRNA
*Staphylococcus epidermidis* (Clinical isolate)	−	−	−
*Corynebacterium Sp.* (Clinical isolate)	−	−	−
*Pseudomonas aeruginosa* (ATCC 27853)	−	−	−
*Enterococcus faecalis* (ATCC 29212)	−	−	−
*Escherichia coli* (ATCC 25922)	−	−	−
*Staphylococcus aureus* (ATCC 29213)	−	−	−
*Proteus sp.* (Clinical isolate)	−	−	−
*Candida albicans* (Clinical isolate)	−	−	−
*Streptococcus pneumoniae* (ATCC 7214)	−	−	−
*Haemophilus influenzae* (ATCC 49247)	−	−	−
*Neisseria meningitidis* (Clinical isolate)	−	−	−
*Helicobacter pylori* (ATCC 700824)	+	+	+
*Helicobacter pylori* (ATCC 700392)	+	+	+

### Real-time PCR

TaqMan PCR was used to amplify fragments of the 16S rRNA and ureA genes, and SyBR Green PCR was used to amplify fragments of the 23S rRNA gene of *H. pylori* and the human β-globin gene. Real-time PCR was carried out in an ABI 7500 thermocycler (Applied Biosystems) with the following thermal cycling conditions:

- TaqMan PCR for the 16S rRNA gene: Initial uracil-DNA glycosylase (UNG) incubation at 37°C for 10 min., followed by denaturation at 95°C for 10 min., and 40 cycles consisting of denaturation at 92°C for 15 s. followed by annealing/extension at 64°C for 1 min.
- TaqMan PCR for the UreA gene: Initial UNG incubation at 37°C for 10 min., followed by denaturation at 95°C for 10 min., and 40 cycles consisting of denaturation at 95°C for 15 s, touchdown annealing from 60°C to 57°C for 10 s (temperature decrements, 0.2°C), and extension at 72°C for 35 s.
- SyBR Green PCR for the 23S rRNA gene of *H. pylori*: Initial UNG incubation at 37°C for 10 min., followed by denaturation at 95°C for 10 min, and 45 cycles consisting of denaturation at 95°C for 30 s, annealing at 60°C for 30 s, and extension at 72°C for 35 s. After amplification, a melting step was performed.

The 25 µl PCR reaction mixture was prepared with 12.5 µl of 2× SensiMix (dU) (Quantace LTD., London, UK), 0.5 µl of 50× UNG, and 2.5 µl of DNA for all genes. MgCl_2_ concentrations were 5.5 mM, 4.0 mM, and 3.0 mM for the 16S rRNA, ureA ,and 23S rRNA genes, respectively. Primer concentrations were 0.1 µM, 0.25 µM, and 0.4 µM for the 16S rRNA, ureA, and 23S rRNA genes, respectively. The TaqMan probe concentration was 4.0 nM for 16S rRNA and 20 nM for ureA.

TaqMan probes were labeled with the fluorescent dyes 6-carboxyfluorescein (6-FAM) on the 5′ end and with 6-carboxytetramethylrhodamine (TAMRA) on the 3′ end; probes were purified by high performance liquid chromatography. Primers and probes were purchased from Stabvida Lda. (St. Antonio de Oeiras, Portugal). Corresponding sequences are listed in Table 3.

**Table 3:** Primers and probes used to amplify the target genes.

Gene	Forward primer	Reverse primer	Length (bp)	Ref.
16S rRNA	5′-ctc att gcg aag gcg acc t-3′	5′-tct aat cct gtt tgc tcc cca-3′	76	[13]
ureA	5′-cgt ggc aag cat gat cca t-3′	5′-ggg tat gca cgg tta cga gtt t-3′	77	[14]
23S rRNA	5′-gga gct gtc tca acc aga gat tc-3′	5′-cgc atg ata ttc cca tta gca g-3′	132	[15]
Human β-globin	5′-aca caa ctg tgt tca cta gc-3′	5′-caa ctt cat cca cgt tca cc-3′	110	[23]

TaqMan probe	Sequence
16S rRNA	5′- (6-FAM)-att act gac gct gat tgc gcg aaa gc-TAMRA- 3′
ureA	5′- (6-FAM)-tca gga aac atc gct tca ata ccc act t-TAMRA-3′

(bp): base pairs.

### Human β-globin amplification

The quality of the DNA isolated from the biopsy specimens was confirmed by an initial PCR. We used PCR on each DNA extract to amplify a 110 bp fragment from the β-globin gene region [23]. All tests were performed by two experienced molecular biologists who were blinded to the *H. pylori* status of the samples and to the results of the other tests.

### Histological predictors of infection and immunohistochemical analysis

Two specialized pathologists (AC,ES) who were unaware of the final *H. pylori* status analyzed the biopsy specimens and recorded the presence of active gastritis (yes/no), chronic gastritis (yes/no), lymphoid aggregates (yes/no), atrophic gastritis (yes/no), and intestinal metaplasia (yes/no). A specific immunohistochemical stain was also performed to ensure that *H. pylori* was not microscopically detectable. A 3 µm-thick section from the same paraffin blocks used for PCR was fixed on adhesive-coated slides. Specimens were incubated with primary antibody (polyclonal rabbit anti-*Helicobacter pylori*) at a dilution 1/500, and biopsies were immunohistochemically stained with *H. pylori* antibodies using the Envision™ Flex/Hrp technique. Primary and secondary antibodies were purchased from Dako (Glostrup, Denmark).

### Statistical analyses

The three PCR methods were validated by PCR analysis of the three PCR primers in the 17 positive controls and in the 32 negative controls.

The prevalence of *H. pylori* infection determined by the different PCR methods is reported as percentage (95% confidence intervals) for the individual PCR techniques and for combinations of two or more techniques. In the 52 patients with further follow-up, we calculated the sensitivity, specificity, positive predictive values (PPV), and negative predictive values (NPV) together with their 95% confidence intervals for the histological parameters and different PCR methods, using the results of the delayed UBT as the gold standard. Sensitivities and specificities were compared using the McNemar test as described by Biswas [24]. We used the chi-square test to analyze differences in proportions between groups. We used SPSS 15.0.1 for Windows (SPSS Inc. Chicago, IL) for all analyses. Significance was set at p<0.05.

## Results

### Species specificity of the PCR assay

All DNA derived from non-*Helicobacter* species was negative for all three PCR products; the two strains of *H. pylori* were positive for all three products. Table 2 summarizes these results.

### Validation of PCR assays

All the antral biopsy specimens from the 17 positive controls were positive on all three PCR assays. All the antral biopsy specimens from the 32 negative controls were negative on all three PCR assays, except those from one patient, which were positive for ureA and those from another patient, which were positive for all three genes thus raising the possibility of occult infection undetected by conventional methods.

All samples were positive for β-globin amplification, indicating that no PCR inhibitors were present.

### Prevalence of infection according to the different methods

Real-time PCR was positive for 16S rRNA in 25 of the 52 patients (48%) and for both UreA and 23S rRNA in 19 of 52 (37%). Immunohistochemical analysis detected the infection in only three (6%) of the patients. The sensitivity, specificity, PPV, and NPV for the PCR assays and immunohistochemical staining in this group with respect to the delayed UBT gold standards are reported in Table 4. We found no association between the number of biopsies in the paraffin block and the rate of *H. pylori* detection by PCR.

**Table 4:** Sensitivity, specificity, positive predictive value (PPV), negative predictive value (NPV), positive likelihood ratio, and negative likelihood ratio of *H. pylori* diagnostic tests in histology-negative PUB patients.

	Sensitivity(%) (95% CI)	Specificity(%) (95% CI)	PPV (%) (95% CI)	NPV (%) (95% CI)	(+) Likelihood ratio	(−) Likelihood ratio
**Immunohistochemistry**	7 (2%–19%)	100 (66%–100%)	100 (31%–99%)	20 (11%–35%)	∞	0.93
**16S rRNA**	55 (39%–70%)	80 (44%–96%)	92 (73%–99%)	30 (14%–50%)	2.75	0.56
**ureA**	43 (28%–59%)	90 (54%–99%)	95 (72%–99%)	29 (15%–48%)	4.3	0.63
**23S rRNA**	41 (26%–57%)	80 (44%–96%)	90 (65%–98%)	24 (12%–43%)	2.05	1.12
**16S rRNA + ureA**	64 (48%–78%)	80 (44%–96%)	93 (76%–99%)	35 (17%–57%)	3.2	0.45
**16S rRNA + 23S rRNA**	60 (43%–74%)	60 (27%–86%)	86 (67%–95%)	26 (11%–49%)	1.5	0.66
**ureA + 23S rRNA**	57 (41%–72%)	70 (35%–92%)	89 (70%–97%)	28 (13%–50%)	1.9	0.61
**16S rRNA + ureA + 23S rRNA**	67 (50%–80%)	60 (27%–86%)	88 (70%–96%)	30 (13%–54%)	1.67	0.55

The sensitivity of all PCR assays was significantly higher than that of immunohistochemical staining, with p values ranging from 0.01 to <0.001. No significant differences were observed, however, between PCR assays. Furthermore, the number of “true” *H. pylori*-negative cases was too low to enable statistical comparison of the specificities. All combinations of PCR tests improved the sensitivity but slightly decreased the specificity (Table 4). All PCR combinations were significantly more sensitive than the ureA PCR test alone (p<0.05) or the 23S rRNA PCR test alone (p<0.05) and marginally better than the 16S rRNA test alone. No significant differences were observed between combinations of PCR tests.

In addition, we evaluated the predictive value of the different histological parameters for *H. pylori* infection (Table 5). The presence of acute gastritis was the only histological parameter with acceptable sensitivity and specificity for detecting active *H. pylori* infection.

**Table 5:** Sensitivity, specificity, positive (PPV) and negative (NPV) predictive values, and positive and negative likelihood ratios of histopathological parameters for the diagnostic of infection in *H. pylori* negative biopsies in PUB patients.

	Sensitivity(%) (95% CI)	Specificity(%) (95% CI)	PPV (%) (95% CI)	NPV (%) (95% CI)	(+) Likelihood ratio	(−) Likelihood ratio
**Acute gastritis**	76 (60%–87%)	80 (44%–96%)	94 (79%–99%)	49 (32%–66%)	3.8	0.3
**Chronic gastritis**	97 (74%–100%)	20 (10%–36%)	83 (47%–97%)	67 (13%–98%)	1.21	0.15
**Lymphoid follicles**	67 (50%–80%)	50 (20%–80%)	85 (67%–94%)	26 (10%–51%)	1.34	0.66
**Atrophy**	50 (34%–65%)	30 (8%–65%)	88 (66%–96%)	25 (11%–45%)	0.71	1.67
**Metaplasia**	36 (19%–56%)	60 (27%–86%)	71 (47%–87%)	13 (4%–31%)	0.9	1.07

## Discussion

We found that real-time PCR detects *H. pylori* in more than 2/3 of the histology-negative sample, being far more sensitive than conventional histological identification of the bacteria or immunohistochemistry and may be a useful alternative for detecting *H. pylori* infection in patients with PUB. The real-time PCR described here has the additional advantage that it can be used in FFPE samples, allowing detection of *H. pylori* in both prospective and retrospective studies.

In addition to showing that PCR is useful in the acute PUB setting, our study gives support to the recently challenged [25] classical view that even a small number of polymorphonuclear neutrophils in a gastric biopsy is an almost certain indicator of active *H. pylori* infection [26]. In PUB patients this affirmation holds true even when the bacteria are not detectable by direct visualization or immunohistochemistry. For this reason, the finding of acute inflammation in biopsies negative for *H. pylori* should prompt additional studies.

Our findings confirm that *H. pylori* infection is elusive in PUB. Studies of *H. pylori* diagnostic methods in patients with PUB have found 25% to 50% false-negative results for histological study of biopsies obtained during bleeding episodes [4], [27]. Consequently, current guidelines recommend either more sensitive diagnostic analysis or delayed testing to rule out the infection [28].

Although there is no fully accepted explanation for the reduced reliability of diagnostic tests for *H. pylori* in the PUB setting, various factors could lead to a transient decrease in bacterial density that would make detection more difficult. First, due to the bactericidal effects of serum, the presence of blood in the stomach would directly lower bacterial density [29], [30]. Second, the accuracy of the rapid urease test in the PUB setting could also be affected by the presence of albumin in the gastric lumen, which could prevent color change by acting as a buffer on the pH indicator [31]. Third, these patients are often taking PPIs, which could reduce the bacterial load [32]. Finally, large-volume gastric rinsing before endoscopy may result in temporary removal of the bacteria [33].

In comparison with other invasive techniques, PCR provides very sensitive and accurate diagnosis of *H. pylori* in patients with non-bleeding peptic ulcers [13], [34]. However, few studies have examined the diagnostic accuracy of PCR in patients with PUB [9], [35], [36]. Most of them indicate that PCR is more sensitive than other tests for *H. pylori* in PUB, though they did not evaluate real-time PCR or use FFPE biopsies. Real-time PCR has several advantages over conventional PCR, such as short working time, high specificity, and low risk of cross contamination. However, because of the diversity of the *H. pylori* genome, the choice of specific primers is of paramount importance. We used PCR for a small part of the 16S rRNA, ureA, and 23S rRNA genes, which have been reported to be highly sensitive and specific and are well preserved in FFPE samples [13]–[15]. Our results indicate that the most accurate PCR test for *H. pylori* in PUB patients is either the combination of 16S rRNA and ureA (sensitivity, specificity, PPV, and NPV of 64%, 80%, 93%, and 35%, respectively) or the combination of all three targets (sensitivity, specificity, PPV, and NPV of 67%, 60%, 88%, and 30%, respectively).

Because real-time PCR is based on DNA detection and does not require viable bacteria, it is a useful adjunct to culturing, the gold standard in the diagnosis of *H. pylori*, especially when antimicrobial treatment has already started [37]. Real–time PCR is inexpensive; indeed, it is less expensive than histology. Thus, the cost of using real-time PCR in patients negative for *H. pylori* at histology will likely be compensated by the clinical benefits derived from detecting the infection in a significant percentage of histologically negative biopsies. Furthermore, PCR techniques have been progressively incorporated into the routine workload of microbiology units, and some PCR tests are already routine in clinical practice. Thus, incorporating real-time PCR tests into the routine work-up for histologically negative biopsies from patients with PUB seems feasible.

The present study casts doubt on the suggestion that the prevalence of *H. pylori* in bleeding ulcers is lower than in non-bleeding ones [3], [38]–[42]. Some studies have even reported a rising prevalence of idiopathic ulcers [43]. However, few of these studies used delayed tests for *H. pylori* or PCR methods. As PCR detected *H. pylori* in more than two-thirds of the histology-negative biopsies in our series, it is reasonable to wonder how many of the “idiopathic” PUB found in previous studies were actually under-diagnosed *H. pylori-*related PUB. The true prevalence of idiopathic ulcers and idiopathic PUB remains to be determined.

Some points of the study deserve specific comment: First, although the sensitivity of PCR (range 41%–67%) may appear low, it is important to take into account that the biopsies studied were negative for *H. pylori* at histology. Thus, not only did PCR detect *H. pylori* in all the histology-positive control biopsies, it also detected *H. pylori* in two-thirds of the histology-negative samples from patients with a positive delayed ^13^C-UBT [16]. Likewise, although the specificity may seem relatively low, there is no perfect gold standard for *H. pylori* and there are no techniques able to detect all cases of infection. Thus, as real-time PCR is highly sensitive, it is quite possible that at least one of the patients considered false-positives of PCR were, in fact, infected and would therefore be false-negatives of the delayed ^13^C-UBT we used as a gold-standard.

This study deals with a selected subpopulation of PUB patients with initially negative histological findings. In our environment, these patients are rare and therefore difficult to recruit. This explains our limited sample size and consequently the wide 95% CI for the sensitivity of PCR (around ±15%). Nevertheless, the sample size is large enough to demonstrate that PCR is far more sensitive than histology or immunohistochemistry and that the best results are observed when two or three genes are targeted.

A final point is that we evaluated only antral biopsy specimens. Gisbert et al. [2] suggested that histological examination of specimens from both the antrum and corpus improved diagnostic sensitivity from 70% to 83%. However, a previous study [16] found no significant benefit for the combination of antral and corpus biopsies. In the present study, no corpus samples were available for PCR analysis, so we could not analyze the potential usefulness of PCR or immunohistochemical study of biopsy specimens from the corpus.

Our results raise diverse questions for further research. The prevalence of *H. pylori* infection is high in southern Europe, both in the general population and in PUB patients [44], [45]; it would be interesting to know whether our results could be confirmed in low-prevalence areas like northern Europe, Canada, or some parts of the United States. It would also be interesting to evaluate whether real-time PCR is useful for detecting *H. pylori* in other scenarios where the infection is often elusive, such as in patients with low-grade gastric MALT lymphoma or gastric cancer or even in those with uncomplicated ulcer or dyspepsia receiving PPI before endoscopy. Finally, it would be interesting to compare real-time PCR with conventional tests in an unselected population of dyspeptic patients.

In conclusion, our study shows that real-time PCR improves the detection of *H. pylori* in FFPE biopsies in PUB patients and can be used to identify *H. pylori* infection in patients with negative histological findings. Our results support previous data that suggest that a significant percentage of “idiopathic” bleeding ulcers may actually be false-negative results of conventional tests to detect *H. pylori*.

## References

[1] Suerbaum S, Michetti P (2002). *Helicobacter pylori* infection.. N Engl J Med.

[2] Gisbert JP, Khorrami S, Carballo F, Calvet X, Gene E (2004). Meta-analysis: *Helicobacter pylori* eradication therapy vs. antisecretory non-eradication therapy for the prevention of recurrent bleeding from peptic ulcer.. Aliment Pharmacol Ther.

[3] Gisbert JP, Pajares JM (2003). *Helicobacter pylori* and bleeding peptic ulcer: what is the prevalence of the infection in patients with this complication?. Scand J Gastroenterol.

[4] Gisbert JP, Abraira V (2006). Accuracy of *Helicobacter pylori* diagnostic tests in patients with bleeding peptic ulcer: a systematic review and meta-analysis.. Am J Gastroenterol.

[5] Raderer M, Streubel B, Wohrer S, Hafner M, Chott A (2006). Successful antibiotic treatment of *Helicobacter pylori* negative gastric mucosa associated lymphoid tissue lymphomas.. Gut.

[6] Bik EM, Eckburg PB, Gill SR, Nelson KE, Purdom EA (2006). Molecular analysis of the bacterial microbiota in the human stomach.. Proc Natl Acad Sci USA.

[7] He Q, Wang JP, Osato M, Lachman LB (2002). Real-time quantitative PCR for detection of *Helicobacter pylori*.. J Clin Microbiol.

[8] Tankovic J, Chaumette-Planckaert MT, Deforges L, Launay N, Le Glaunec JM (2007). Routine use of real-time PCR for detection of *Helicobacter pylori* and of clarithromycin resistance mutations.. Gastroenterol Clin Biol.

[9] Lo CC, Lai KH, Peng NJ, Lo GH, Tseng HH (2005). Polymerase chain reaction: a sensitive method for detecting *Helicobacter pylori* infection in bleeding peptic ulcers.. World J Gastroenterol.

[10] Ciesielska U, Jagoda E, Marciniak Z (2002). Value of PCR technique in detection of *Helicobacter pylori* in paraffin-embedded material.. Folia Histochem Cytobiol.

[11] Scholte GH, van Doorn LJ, Quint WG, Lindeman J (1997). Polymerase chain reaction for the detection of *Helicobacter pylori* in formaldehyde-sublimate fixed, paraffin-embedded gastric biopsies.. Diagn Mol Pathol.

[12] Weiss J, Mecca J, da Silva E, Gassner D (1994). Comparison of PCR and other diagnostic techniques for detection of *Helicobacter pylori* infection in dyspeptic patients.. J Clin Microbiol.

[13] Kobayashi D, Eishi Y, Ohkusa T, Ishige, Suzuki T (2002). Gastric mucosal density of *Helicobacter pylori* estimated by real-time PCR compared with results of urea breath test and histological grading.. J Med Microbiol.

[14] Schabereiter-Gurtner C, Hirschl AM, Dragosics B, Hufnagl P, Puz S (2004). Novel real-time PCR assay for detection of *Helicobacter pylori* infection and simultaneous clarithromycin susceptibility testing of stool and biopsy specimens.. J Clin Microbiol.

[15] Lascols C, Lamarque D, Costa JM, Copie-Bergman C, Le Glaunec JM (2003). Fast and accurate quantitative detection of *Helicobacter pylori* and identification of clarithromycin resistance mutations in *H. pylo*ri isolates from gastric biopsy specimens by real-time PCR.. J Clin Microbiol.

[16] Guell M, Artigau E, Esteve V, Sanchez-Delgado J, Junquera F (2006). Usefulness of a delayed test for the diagnosis of *Helicobacter pylori* infection in bleeding peptic ulcer.. Aliment Pharmacol Ther.

[17] Castro-Fernandez M, Sanchez-Munoz D, Garcia-Diaz E, Miralles-Sanchiz J, Vargas-Romero J (2004). Diagnosis of *Helicobacter pylori* infection in patients with bleeding ulcer disease: rapid urease test and histology.. Rev Esp Enferm Dig.

[18] Gisbert JP, Esteban C, Jimenez I, Moreno-Otero R (2007). 13C-urea breath test during hospitalization for the diagnosis of *Helicobacter pylori* infection in peptic ulcer bleeding.. *Helicobacter*.

[19] Gisbert JP, Trapero M, Calvet X, Mendoza J, Quesada M (2004). Evaluation of three different tests for the detection of stool antigens to diagnose *Helicobacter pylori* infection in patients with upper gastrointestinal bleeding.. Aliment Pharmacol Ther.

[20] Calvet X, Lario S, Ramírez-Lázaro MJ, Montserrat A, Quesada M, Reeves L (2010). Comparative accuracy of 3 monoclonal stool tests for diagnosis of *Helicobacter pylori* infection among patients with dyspepsia.. Clin Infect Dis.

[21] Calvet X, Sánchez-Delgado J, Montserrat A, Lario S, Ramírez-Lázaro MJ (2009). Accuracy of diagnostic tests for *Helicobacter pylori*: a reappraisal.. Clin Infect Dis.

[22] Chomczynski P, Sacchi N (1987). Single-step method of RNA isolation by acid guanidinium thiocyanate-phenol-chloroform extraction.. Anal Biochem.

[23] Saiki RK, Bugawan TL, Horn GT, Mullis KB, Erlich HA (1986). Analysis of enzymatically amplified beta-globin and HLA-DQ alpha DNA with allele-specific oligonucleotide probes.. Nature.

[24] Biswas B (2006). Assessing agreement for diagnostic devices.. http://www.amstat.org/meetings/fdaworkshop/presentations/2006/Assessing%20Agreement%20for%20diagnostic%20tests%202006.ppt.

[25] Genta RM, Lash RH (2010). Helicobacter pylori-negative gastritis: Seek, yet ye shall not always find.. Am J Surg Pathol.

[26] Dixon MF, Genta RM, Yardley JH, Correa P (1994). Classification and grading of gastritis. The updated Sydney System. International Workshop on the Histopathology of Gastritis, Houston 1994.. Am J Surg Pathol.

[27] Calvet X, Barkun AN, Kuipers EJ, Lanas A, Bardou M (2009). Is *H. pylori* testing clinically useful in the acute setting of upper gastrointestinal bleeding? A systematic review.. Gastroenterology.

[28] Lanas A, Calvet X, Feu F, Ponce J, Gisbert JP (2010). First Spanish consensus on peptic ulcer bleeding management. Consenso sobre Hemorragia Digestiva por Úlcera Péptica.. Med Clin (Barc).

[29] Martin de Argila C, Boixeda D (2001). Practical considerations for the diagnosis of *Helicobacter pylori* infection [in Spanish].. Med Clin (Barc).

[30] Houghton J, Ramamoorthy R, Pandya H, Dhirmalani R, Kim KH (2002). Human plasma is directly bacteriocidal against *Helicobacter pylori in vitro*, potentially explaining the decreased detection of *Helicobacter pylori* during acute upper GI bleeding.. Gastrointest Endosc.

[31] Leung WK, Sung JJ, Siu KL, Chan FK, Ling TK (1998). False-negative biopsy urease test in bleeding ulcers caused by the buffering effects of blood.. Am J Gastroenterol.

[32] Graham DY, Opekun AR, Hammoud F, Yamaoka Y, Reddy R (2003). Studies regarding the mechanism of false negative urea breath tests with proton pump inhibitors.. Am J Gastroenterol.

[33] Fantry GT, Rosenstein AH, James SP (1999). Confounding factors in the detection of *Helicobacter pylori* infection in patients with upper gastrointestinal bleeding.. Am J Gastroenterol.

[34] Lage AP, Godfroid E, Fauconnier A, Burette A, Butzler JP (1995). Diagnosis of *Helicobacter pylori* infection by PCR: comparison with other invasive techniques and detection of cagA gene in gastric biopsy specimens.. J Clin Microbiol.

[35] Lin HJ, Lo WC, Perng CL, Tseng GY, Li AF (2005). Mucosal polymerase chain reaction for diagnosing *Helicobacter pylori* infection in patients with bleeding peptic ulcers.. World J Gastroenterol.

[36] Castillo-Rojas G, Ballesteros MA, Ponce de LS, Leon S (2002). Bleeding peptic ulcers and presence of *Helicobacter pylori* by various tests: a case-control study.. Eur J Gastroenterol Hepatol.

[37] Resti M, Micheli A, Moriondo M, Becciolini L, Cortimiglia M (2009). Comparison of the effect of antibiotic treatment on the possibility to diagnose invasive pneumococcal disease by cultural or molecular methods: a prospective observational study of children with proven pneumococcal infection.. Clin Therapeutics.

[38] Lee JM, Breslin NP, Fallon C, O'Morain CA (2000). Rapid urease tests lack sensitivity in *Helicobacter pylori* diagnosis when peptic ulcer disease presents with bleeding.. Am J Gastroenterol.

[39] Wu CY, Poon SK, Chen GH, Chang CS, Yeh HZ (1999). Interaction between *Helicobacter pylori* and non-steroidal anti-inflammatory drugs in peptic ulcer bleeding.. Scand J Gastroenterol.

[40] Sotoudehmanesh R, Asgari AA, Fakheri HT, Nouraie M, Khatibian M (2005). Peptic ulcer bleeding: is *Helicobacter pylori* a risk factor in an endemic area?. Indian J Gastroenterol.

[41] Hayat M, Everett S, Breslin NP, Moayyedi P, O'Mahony S (1999). The prevalence of *Helicobacter pylori* in bleeding peptic ulcer disease.. Gut.

[42] Pilotto A, Leandro G, Di Mario F, Franceschi M, Bozzola L (1997). Role of *Helicobacter pylori* infection on upper gastrointestinal bleeding in the elderly: a case-control study.. Dig Dis Sci.

[43] Hung LC, Ching JY, Sung JJ, To KF, Hui AJ (2005). Long-term outcome of *Helicobacter pylori*-negative idiopathic bleeding ulcers: a prospective cohort study.. Gastroenterology.

[44] Arroyo MT, Forne M, de Argila CM, Feu F, Arenas J (2004). The prevalence of peptic ulcer not related to *Helicobacter pylori* or non-steroidal anti-inflammatory drug use is negligible in southern Europe.. *Helicobacter*.

[45] Gisbert JP, Gonzalez L, de Pedro A, Valbuena M, Prieto B (2001). *Helicobacter pylori* and bleeding duodenal ulcer: prevalence of the infection and role of non-steroidal anti-inflammatory drugs.. Scand J Gastroenterol.

